# Blood-Brain Barrier Effects of the *Fusarium* Mycotoxins Deoxynivalenol, 3 Acetyldeoxynivalenol, and Moniliformin and Their Transfer to the Brain

**DOI:** 10.1371/journal.pone.0143640

**Published:** 2015-11-23

**Authors:** Matthias Behrens, Sabine Hüwel, Hans-Joachim Galla, Hans-Ulrich Humpf

**Affiliations:** 1 Institute of Food Chemistry, Westfälische Wilhelms-Universität Münster, Corrensstr. 45, 48149, Münster, Germany; 2 Institute of Biochemistry, Westfälische Wilhelms-Universität Münster, Wilhelm-Klemm-Str. 2, 48149, Münster, Germany; Hungarian Academy of Sciences, HUNGARY

## Abstract

**Background:**

Secondary metabolites produced by *Fusarium* fungi frequently contaminate food and feed and have adverse effects on human and animal health. *Fusarium* mycotoxins exhibit a wide structural and biosynthetic diversity leading to different toxicokinetics and toxicodynamics. Several studies investigated the toxicity of mycotoxins, focusing on very specific targets, like the brain. However, it still remains unclear how fast mycotoxins reach the brain and if they impair the integrity of the blood-brain barrier. This study investigated and compared the effects of the *Fusarium* mycotoxins deoxynivalenol, 3-acetyldeoxynivalenol and moniliformin on the blood-brain barrier. Furthermore, the transfer properties to the brain were analyzed, which are required for risk assessment, including potential neurotoxic effects.

**Methods:**

Primary porcine brain capillary endothelial cells were cultivated to study the effects of the examined mycotoxins on the blood-brain barrier *in vitro*. The barrier integrity was monitored by cellular impedance spectroscopy and ^14^C radiolabeled sucrose permeability measurements. The distribution of the applied toxins between blood and brain compartments of the cell monolayer was analyzed by high performance liquid chromatography-mass spectrometry to calculate transfer rates and permeability coefficients.

**Results:**

Deoxynivalenol reduced the barrier integrity and caused cytotoxic effects at 10 μM concentrations. Slight alterations of the barrier integrity were also detected for 3-acetyldeoxynivalenol. The latter was transferred very quickly across the barrier and additionally cleaved to deoxynivalenol. The transfer of deoxynivalenol and moniliformin was slower, but clearly exceeded the permeability of the negative control. None of the compounds was enriched in one of the compartments, indicating that no efflux transport protein is involved in their transport.

## Introduction

### The blood-brain barrier

The blood-brain barrier (BBB) is a highly selective permeability barrier separating the blood stream from the interstitial fluid in the brain. This barrier controls the nutrient supply for all cerebral cells and protects the highly sensitive neurons from any adverse effects caused by changes in osmotic pressure or xenobiotics as well as several endogenous compounds present in the bloodstream. The BBB mainly consists of three cell types: Endothelial cells, directly forming the cerebral microvessels to control the efflux and influx of nutrients and xenobiotics [1]. They are coated by a basal lamina, in which pericytes are located. Pericytes exhibit various regulatory functions, synthesize most components of the basal lamina and play a key role in BBB development [2, 3]. Astrocytes contribute to the microstructure of the brain and also have regulatory functions by various interactions with the endothelial cells [4]. The astrocyte endfeet are located around the basal lamina. Furthermore, astrocytes synthesize cholesterol, which is the main component of the myelin sheath, the electrical insulator of neuronal axons [5].

The efficient barrier function of the BBB is achieved by the combination of three specific barrier properties. The first so called physical barrier hinders polar compounds from paracellular diffusion through the gaps between the endothelial cells. It is formed by the high levels of tight junctions and their transmembrane proteins. Occludin, which is connected to the *zonula occludens* proteins 1, 2 and 3, regulates the tight junctions, whereas the claudins 3, 5 and 12 are mainly responsible for the actual barrier and elevated transendothelial electrical resistances of usually more than 1000 Ω cm^2^ (TEER) [4].

Small, lipophilic molecules are capable of permeating the cellular membranes by diffusion. To prevent these compounds from entering neuronal tissues, endothelial cells exhibit a large variety of transport proteins, which form a second barrier, controlling the influx and especially the efflux of these substances. Transport proteins also play a key role in multidrug resistance (MDR) by protecting cancer cells through the export of therapeutics out of the cells, making for example a treatment of cancer difficult [4].

The third barrier system is called metabolic barrier. It is formed by the phase I and phase II metabolic enzymes of endothelial cells. Conjugation of substrates with sulfate, glucuronic acid or glutathione results in higher water solubility. After exporting these conjugates via the basolateral membrane out of the endothelial cells, they are unlikely to permeate cellular membranes and can therefore be excreted easier from the bloodstream [4, 1].

Primary porcine brain capillary endothelial cells (PBCEC) form a well characterized, flexible model to study interactions of pericytes [6] and astrocytes [7] with endothelial cells. Furthermore, they have been successfully used for transport studies of xenobiotics [8, 9]. One of the biggest advantages of PBCEC are the high TEER values of more than 1000 Ω cm^2^ that can be obtained even in monocultures *in vitro*. This restrictive barrier shows good correlation with the BBB *in vivo*. Additionally, physiological concentrations of hydrocortisone are used to improve the TEER values. Hydrocortisone enhances the tightness of the barrier by cytoskeletal rearrangements [10, 11].

In comparison to other models, PBCEC combine many advantages of handling cell cultures compared to *in vivo* and *ex vivo* studies like reduced interindividual differences and no need of invasive methods. Compared to cell lines, primary cells like PBCEC maintain more morphological features of the brain endothelium [3]. Unlike cell lines, primary cells need to be harvested, purified and monitored for contamination more frequently. This causes higher expenses and results in more laborious protocols, required for individual cell culture experiments. However, when comparing static cell culture models, like PBCEC, to the *in vivo* situation, the latter not only includes more cell types in the microenvironment, but also the dynamic blood circulation in the body. The shear stress caused by the constant blood flow is known to improve polarization and differentiation of endothelial cells at the BBB [12].

The human cerebral microvascular endothelial cell line hCMEC/D3 is also known to show good correlation with the human BBB phenotype, but their TEER values rarely exceed 50 Ω cm^2^ [13, 14]. This enables transport studies only for a short period of time, because the diffusion through the endothelial cell monolayer is faster and not completely hindered. A recent publication used a targeted proteomic HPLC-MS/MS analysis to compare the transmembrane protein distribution of hCMEC/D3 with human brain microvessels. Although most levels of these transmembrane proteins were comparable, when looking at ATP-binding cassette transporters, ABCA8 could not be quantified in hCMEC/D3, whereas ABCA3, ACBA6, MRP1, which are present in hCMEC/D3, were below the limits of quantification in human brain microvessels [15].

In a recent publication, effects of ergot alkaloids produced by fungi of the *Claviceps* genus were shown to easily permeate the BBB *in vitro* [16]. Strong cytotoxic effects of the type A trichothecene mycotoxins T-2 toxin and HT-2 toxin, often detected in *Fusarium*, were observed using the same model as presented in this study. Both compounds were also shown to cross the BBB [17]. Furthermore, T-2 toxin causes severe cytotoxic effects with IC_50_ values of 24±4.5 nM on normal human astrocytes (NHA) [18].

### *Fusarium* mycotoxins

**Fig 1:**
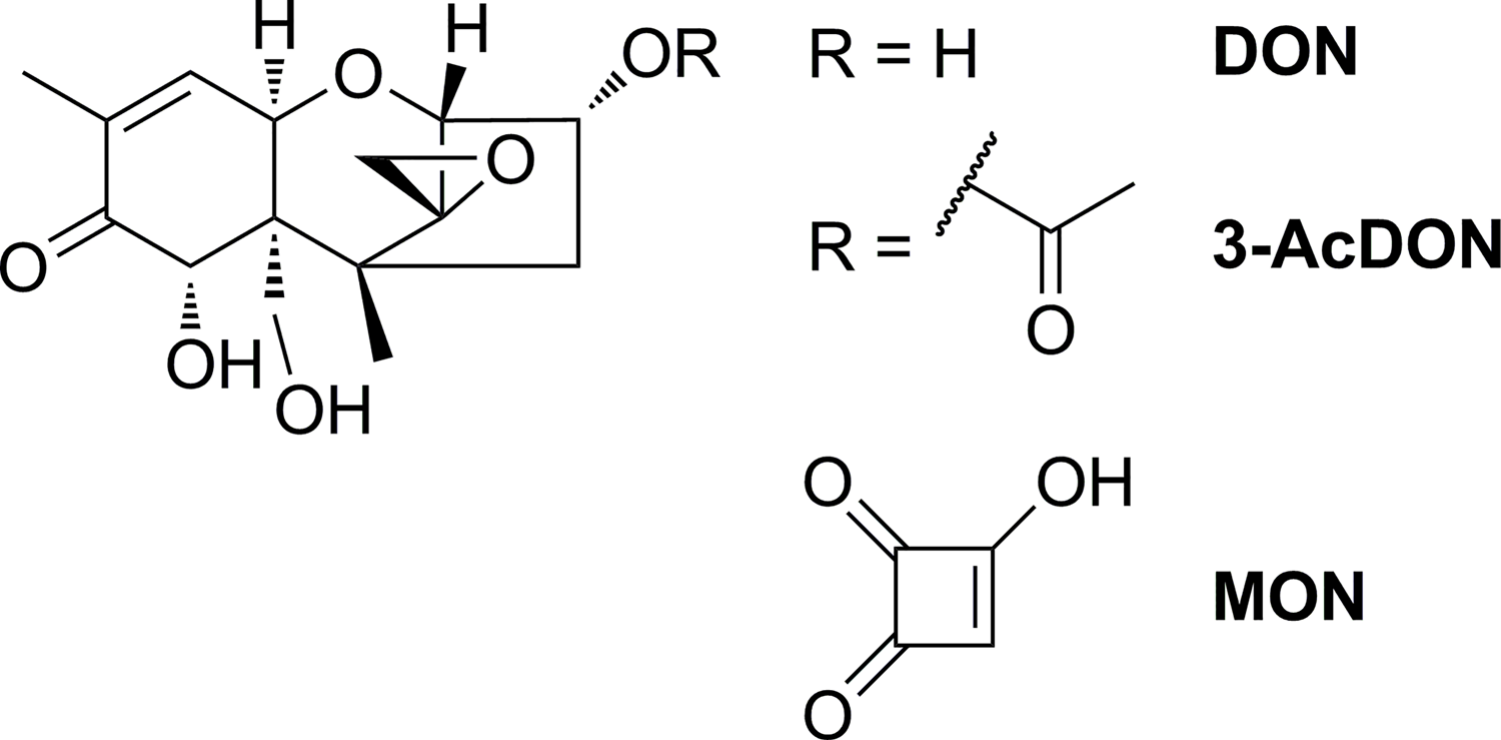
Chemical structures of deoxynivalenol (DON), 3-acetyldeoxynivalenol (3-AcDON) and moniliformin (MON).

For this study, three mycotoxins were chosen according to their occurrence in food and feed, toxicological relevance and lipophilicity. Due to the strong effects of T-2 toxin on the BBB *in vitro*, the effects of other trichothecenes were investigated in this study. Therefore, the type B trichothecenes, deoxynivalenol (DON) and 3-acetyldeoxynivalenol (3-AcDON), which are both frequently detected in grains and cereals, were analyzed. Due to its small molecular size and very high polarity, moniliformin (MON) is hard to analyze and little is known about its toxic effects. MON is an interesting subject for investigating effects on the BBB, because small molecules like MON are more likely to diffuse through cellular membranes. The chemical structures of the three mycotoxins applied on the PBCEC *in vitro* model presented in this study are shown in Fig 1.

The effects of DON on cellular barrier functions have already been studied in intestinal barrier models. DON decreased the transepithelial resistance of Caco-2 monolayers and intestinal epithelial cells and induced inflammation markers such as mitogen-activated protein kinases (MAPK). MAPK reduce the expression of claudin-3 and claudin-4, which are major tight junction proteins in the small intestine. The reduction of claudins contributes to the impairment of the barrier integrity caused by DON in Caco-2 and intestinal epithelial cells [19, 20]. The addition of inhibitors revealed that the DON transfer is neither affected by P-glycoprotein (Pgp) nor by the multidrug resistance-associated proteins (MRPs). Therefore, it was suggested that DON passes the small intestine by the paracellular or perhaps the transcellular pathway [21]. Due to the toxic effects of DON, the European Food Safety Authority (EFSA) and the Joint FAO/WHO Expert Committee on Food Additives (JECFA) established a tolerable daily intake (TDI) and provisional maximal tolerable daily intake (PMTDI) for DON of 1 μg/kg body weight per day [22, 23]. The human exposure to DON and DON-glucuronides was assessed in HPLC-MS/MS based urinary biomarker studies and compared to the TDI and PMTDI. The calculated provisional daily intake (PDI) of 12% of the participants in a German study exceeded the TDI and PMTDI [24]. In an Austrian study one third of the calculated PDI exceeded the TDI [25], whereas 48% of the calculated PDI in a Croatian study exceeded the TDI [26]. Similar results were obtained in animal studies. The administration of DON via naturally contaminated corn to pigs was used to calculate a mean oral bioavailability of 54% (acute dose) and 89% (chronic dietary exposure) [27]. Additionally, a very rapid absorption of intergastric administered ^14^C DON (0.6 mg) was observed in pigs. The resulting systemic bioavailability was 48–65% [28]. A similar approach showed that ^14^C DON could also be detected in the cerebrospinal fluid (CSF) after intergastric administration to pigs. The concentration profiles in CSF and blood are very similar and reached their maxima after 4.5 h [29]. Until today this is the only evidence that DON might reach neuronal tissues. Nevertheless, it remained unclear, whether DON, besides permeating the blood-CSF barrier, is also capable of passing the BBB. Furthermore, it is unknown, whether DON is transferred through the intact blood-CSF barrier or leads to a temporary barrier impairment allowing the transfer of endogenous and exogenous molecules to the brain, which would be excluded by an intact barrier. The toxicokinetics of DON were recently reviewed with special regard to the effects of DON to the brain [30]. In addition to the already discussed effects on the small intestine, the alteration of endocrine functions was discussed in this review. The presented risk evaluation revealed that some effects *in vivo* on brain, intestine and immune system could be triggered by DON concentrations close to the TDI [30]. Taken together, these findings indicate that DON itself is transferred to the brain and acts directly on cerebral cells [31]. Furthermore, the effects of DON on the viability of glial cells, which are located within the brain, were studied. DON reduced the viability of microglia stronger compared to astrocytes [32].

3-AcDON is often co-occurring in DON contaminated food and feed. Experiments with intestinal epithelial cells revealed that, compared to DON and 15-AcDON, 3-AcDON is less capable of activating MAPK. Therefore it does not reduce claudin-3 and claudin-4 expression as strong as DON and 15-AcDON and has weaker effects on the transepithelial electrical resistance and intestinal barrier integrity [20]. 3-AcDON showed less cytotoxic effects compared to DON and 15-AcDON in Caco-2 and IPEC-1 cells. Moreover, these three trichothecenes act synergistic in both cell types [33, 34]. 3-AcDON was cleaved to DON to a high extent after oral administration to pigs. Almost no 3-AcDON was recovered in blood, plasma, urine and faeces. Nevertheless, the toxicokinetic fate of 3-AcDON in humans is not well studied and improved analytical methods might be more suitable to detect traces of non-cleaved 3-AcDON [35]. The effects of 3-AcDON were investigated in the present study, because of its differences in polarity and cytotoxicity compared to DON.

Due to its conjugated structure, allowing the delocalization of a negative charge, MON is a strong organic acid and a highly polar compound. Its low molecular weight and small size make it hard to quantify even when using modern analytical methods [36]. However, little is known about the toxicity of MON. In early studies it was observed that MON interacts with the tricarboxylic acid cycle *in vitro*. Concentrations lower than 5 μM inhibit pyruvate and α-ketoglutarate dehydrogenases in isolated rat liver mitochondria. This indicates strong effects on the energy metabolism [37]. A recent study addressing the acute oral toxicity of MON in Sprague-Dawley rats, determined the LD_50_ cut-off value of 25 mg/kg body weight. This is within the same order of magnitude as T-2 toxin, the *Fusarium* mycotoxin considered to exhibit the highest acute toxicity. After 6 h 38% of the administered MON dose was detected in urine and the total recovery reached 42% in urine and less than 1% in feces, indicating a considerably high bioavailability. Non-recovered MON might be metabolized or accumulated in the rat organs [38]. A similar study aimed for the subchronic oral toxicity of MON in Sprague-Dawley rats and proposed a lowest observed adverse effect level (LOAEL) of 3 mg/kg body weight. Between 20.2% and 31.5% of the administered MON dose are excreted via urine, whereas 2% were detected in feces and no indications of MON accumulation have been found [39]. Studies investigating the genotoxicity of MON revealed that MON is clastrogenic in primary rat hepatocytes, but not mutagenic using the Ames test [40]. MON does not reduce the viability of macrophages up to 80 μM. This concentration led to a 20% reduction of cellular viability in dendric cells and is supposed to decreas**e** the immune response after infections [41].

This study focused on *Fusarium* mycotoxins frequently detected in food and feed and their potential abilities to cross the BBB or impair BBB integrity, which are a perquisites to whether or not they might cause adverse effects in the brain. Neurotoxicity is a complex field of research and is often confused with cytotoxic effects to neurons. Neurons are protected by the BBB, in order to exclude cytotoxic compounds in the blood stream from the brain. Therefore, an aim of our study was to evaluate whether DON, 3-AcDON, or MON show adverse effects on the BBB and cause an increased permeation of potentially harmful compounds to the brain. Furthermore, it was evaluated, whether and how fast these mycotoxins cross the intact BBB, to gather data about the cerebral exposure to DON, 3-AcDON and MON. These data are required to assess the potential neurotoxicity of mycotoxins.

## Materials and Methods

### Chemicals and Reagents

All chemicals for mycotoxin quantification were purchased at VWR International GmbH (Darmstadt, Germany), Grüssing GmbH Analytica (Filsum, Germany) and Sigma-Aldrich Chemie GmbH (Steinheim, Germany). Cell culture media and supplements were purchased at Biochrom AG (Berlin, Germany) and PAA Laboratories GmbH (Pasching, Austria). Water was purified by a Milli-Q Gradient A10 system (Merck KGaA, Darmstadt, Germany).

DON and 3-AcDON with purities of ≥95% were isolated as previously described [42]. MON was synthesized as previously described with LC-UV (260 nm) purity of 99% [43]. T-2 toxin with >90% purity (^1^H NMR) was isolated as described [44]. For the following experiments 1 mM stock solutions of each mycotoxin were prepared in acetonitrile (ACN) and stored at -20°C.

### Viability test

To ensure that the applied mycotoxins show no cytotoxic effects, PBCEC were incubated with different concentrations of each toxin. Cellular viability was tested after 48 h using the cell counting kit 8 (CCK-8, Donjindo Laboratories, Tokyo, Japan).

General procedure for cultivation of PBCEC is based on an earlier protocol [9]. For the experiment cryopreserved PBCEC were thawed in a 37°C water bath and transferred to a 50 mL centrifugation tube. After gentle resuspension in complete medium (Medium 199 Earle’s with 100 U/mL penicillin, 100 μg/mL streptomycin, 100 μg/mL gentamycin, 4.1 mM L-glutamine and 10% fetal calf serum) and centrifugation at 220 *g* at 20°C for 10 min the supernatant was removed. Fresh complete medium was added and the cell pellet was gently resuspended twice to a final concentration of 150 000 cells/mL. 100 μL of this suspension were seeded on rat tail collagen coated 96-well plates resulting in 15 000 cells per well. PBCEC were incubated at 37°C, 5% CO_2_ in a saturated humidified atmosphere. After 48 h the medium was replaced with serum-free medium (DMEM/Ham’s F-12 with 100 U/mL penicillin, 100 μg/mL streptomycin, 100 μg/mL gentamycin, 0.7 mM L-glutamine and 550 nM hydrocortisone (Sigma-Aldrich, Steinheim, Germany)). 96 h after seeding 50 μL of the medium were removed and replaced with 50 μL double concentrated mycotoxin solution to achieve final concentrations of 10 nM to 10 μM with a maximum ACN concentration of 1%. After 48 h incubation with the toxin, 10 μL CCK-8 solution were added to each well and incubated for 70 min at 37°C, 5% CO_2_ in a saturated humidified atmosphere according to the manufacturers manual. After the incubation, the absorbance of the formed formazan dye was analyzed at 457 nm with 650 nm as a reference using an Infinite M200 PRO microplate reader with Tecan i-control software version 1.7.1.12 (Tecan, Crailsheim, Germany). The reference absorption was subtracted from the formazan absorption. Afterwards a blank absorption without cells was subtracted. Viabilities are normalized to a solvent adjusted negative control, which was not incubated with any mycotoxin.

### Transfer studies

The thawing and seeding procedure was performed according to the protocol already described for the viability test. Instead of using 96-well plates, 500 μL PBCEC suspension (500 000 cells/mL) were seeded in the upper (apical) compartment of a 12-well Transwell^®^ polycarbonate filter membrane (Corning, Wiesbaden, Germany) with a growth area of 1.13 cm^2^ (250 000 cells/filter). The lower (basolateral) compartment was filled with 1.5 mL complete medium. Medium was exchanged with serum-free medium after 48 h as described before. 96 h after seeding the volume of the apical compartment was filled up to 760 μL. The Transwell^®^ filters were briefly transferred to the cellZscope^®^ cellular impedance spectroscope (nanoAnalytics, Münster, Germany) equipped with a 24 12-well Transwell^®^ filter setup. Each well was previously filled with 1650 μL serum-free medium. Transendothelial electrical resistances (TEER) were analyzed using the cellZscope^®^ device. Only Transwell^®^ filters with TEER values of more than 600 Ω cm^2^ and electrical capacitances (c_CL_) of 0.4 to 0.6 μF/cm^2^ were used for transfer studies. 76 μL of the medium in the apical compartment were exchanged with 76 μL of tenfold concentrated mycotoxin solution. The final concentration of the mycotoxins was 10 μM (for DON additionally 1 μM) at the beginning of the mycotoxin incubation. The final solvent concentration was 1% ACN. Incubation began with 760 μL medium in the apical and 1650 μL medium in the basolateral compartment. To quantify the amount of the transferred mycotoxins, samples were collected 1, 2.5, 6.5, 18, 24, 28, 42 and 48 h after toxin application. To maintain the 1:2.17 ratio of the compartments, the apical samples were 46 μL whereas 100 μL samples were collected from the basolateral compartment. After 48 h the experiment was stopped, and the polycarbonate filter membranes were cut out of the Transwell^®^ inserts with a scalpel and extracted with ACN/H_2_O (4+1, v/v) in an ultrasonic bath for 1 h. The extract was dried under nitrogen atmosphere and reconstituted in ACN/H_2_O (1+9, v/v). The collected samples and filter extracts were analyzed and quantified using high performance liquid chromatography-tandem mass spectrometry (HPLC-MS/MS) or high performance liquid chromatography-high resolution mass spectrometry (HPLC-HRMS) respectively. Transfer rates were calculated as a fraction of the measured amount in the basolateral compartment and the total amount, which was incubated and the beginning of the experiment in the apical compartment.

### Active transport studies

To study the active transfer properties, the cells were treated as for the normal transfer studies, but the mycotoxins were incubated on the apical and basolateral side in equimolar 200 nM concentrations. The changes in concentration were monitored continuously using HPLC-MS/MS or HPLC-HRMS.

### Barrier integrity

#### Cellular impedance spectroscopy

Endothelial cells form tight junctions, which minimize the paracellular diffusion and act like an ohmic resistor increasing the TEER. Therefore, the TEER measurement is a reliable tool to analyze the integrity and tightness of a cellular monolayer. During transfer and active transfer studies, the integrity of the BBB model was monitored using the cellZscope^®^ cellular impedance spectrometer. After the mycotoxins were added to the cell culture medium, the TEER was analyzed until the end of the experiment after 48 h. The wait time after each run was set to 10 min, resulting in 60 scans per 48 h. The results were normalized to the TEER values of the first impedance spectrum of each well. The data were processed using cellZscope 1.3.4 software (nanoAnalytics, Münster, Germany).

#### ^14^C sucrose permeability

To confirm the TEER values obtained by cellular impedance spectroscopy, the Transwell^®^ inserts were tested for their ^14^C sucrose permeability, which is no transporter substrate and does not penetrate cellular membranes.

After 48 h exposure to the mycotoxins 1 μCi (37 kBq) ^14^C sucrose was applied to the apical compartment. Samples from the basolateral compartment were collected after 10, 20, 30, 40, 60 and 80 min, diluted in scintillation fluid, analyzed with a scintillation counter and calculated as previously described [8].

### Mycotoxin quantification

The quantification of DON, 3-AcDON, by HPLC-MS/MS was carried out using an 1100 series (Agilent Technologies, Santa Clara, USA) LC and API 4000 QTrap (AB SCIEX Germany GmbH, Darmstadt, Germany) mass spectrometer. Both devices were operated with Analyst 1.4.2 software (AB SCIEX Germany GmbH, Darmstadt, Germany). For chromatographic separation of DON and 3-AcDON a 100 mm × 2.1 mm Kinetex 2,6u PFP 100 A (Phenomenex, Aschaffenburg, Germany) column equipped with a KrudKatcher Ultra filter (Phenomenex, Aschaffenburg, Germany) was used and maintained at 60°C. Solvent A was ACN+1% formic acid (FA), solvent B H_2_O+1% FA at a flow rate of 100 μL/min. The gradient started with 10% A and, ramped to 80% A in 7 min and held for 1.5 min. At 8.6 min A was set to starting conditions and equilibrated for 11.4 min. An injection volume of 10 μL was used. Turbo V ion source was heated to 450°C with activated interface heater. Further conditions were curtain gas (CUR) 30 psi N_2_, collision gas (CAD) 5 × 10^−5^ torr N_2_, nebulizer gas (GS1) 35 psi N_2_, heater gas (GS2) 45 psi N_2_ and ion spray voltage 4200 V. Declustering potential was 56 V, entrance potential was set to 10 V and dwell times of 50 ms per MRM transition were applied. The MRM transitions for DON were *m/z* 297.2 to *m/z* 203.1 with a collision energy of 12 V, a cell exit potential of 12 V and *m/z* 297.2 to *m/z* 249.1 with a collision energy of 17 V, a cell exit potential of 18. For 3-AcDON they were *m/z* 339.2 to *m/z* 203.1 with a collision energy of 25 V, a cell exit potential of 14 V and *m/z* 339.2 to *m/z* 231.1 with a collision energy of 17 V and a cell exit potential of 18 V.

Due to its small molecular size MON shows only one product ion, which is insufficient for a sound HPLC-MS/MS analysis. Furthermore, the signal/noise ratio of a mass spectrometer at mass/charge ratios of below *m/z* 100 (MON: *m/z* 97) is very low. To compensate these effects, an improved HPLC-HRMS method was used. The high resolution greatly improves the needed selectivity, which could not be provided by tandem mass spectrometers [36]. For chromatography an Accela LC (Thermo Fisher Scientific GmbH, Dreieich, Germany) with Accela Pump 60057-60010 and Accela Autosampler 60057-60020 was used. It was coupled to an LTQ-Orbitrap-XL mass spectrometer (Thermo Fisher Scientific GmbH, Dreieich, Germany). The separation was carried out on a 250 mm x 4.6 mm Synergi 4u Hydro-RP 80A (Phenomenex, Aschaffenburg, Germany) column equipped with a 4.6 mm Fusion-RP pre-column (Phenomenex, Aschaffenburg, Germany) at 40°C. Solvent A was MeOH+1% FA, solvent B H_2_O+1% FA. Separation was performed by isocratic elution with 35% A at a flow rate of 350 μL/min. 10 μL of each sample were injected. The heated electrospray ionization (HESI) source was set to 350°C APCI vaporizer temperature, capillary temperature was 250°C, sheath gas was 50 arbitrary units and aux gas 10 arbitrary units. The instrument was operated in negative ionization mode at a source voltage of -3500 V with a capillary voltage of -35 V and tube lens set to -110 V. A full scan experiment from *m/z* 95 to *m/z* 100 was performed with a resolution of 15 000. The maximum injection time was set to 100 ms and limited to 200 000 ions. For quantification the mass/charge ratio of *m/z* 96.9931±10 ppm was used.

To quantify the mycotoxin distribution between both compartments in transfer studies eight calibration standards ranging from 50 nM to 10 μM diluted in preincubated PBCEC serum free medium with 550 nM hydrocortisone were analyzed at least three times per analysis of one cell preparation. For active transfer studies five standards ranging from 100 nM to 300 nM were diluted. DON and 3-AcDON data were processed with Analyst 1.6.2 software (AB SCIEX Germany GmbH, Darmstadt, Germany). MON data were processed with Xcalibur 2.0.7 SP1 (Thermo Fisher Scientific GmbH, Dreieich, Germany).

### Permeability calculations

To compare the BBB permeation of different compounds, time independent permeability coefficients were calculated. The permeability coefficients *p* were calculated according to Eq 1:

$$p[\text{cm}/\text{s}]=\frac{c_{\text{bas}}[\%]}{c_{0\;\text{h},\text{ap}}[\%]}\cdot \frac{V_{\text{ap}}[{\text{cm}}^{3}]}{A[{\text{cm}}^{2}]\cdot t[\text{s}]}$$ (1)

Where *c*_bas_ is the amount of transferred mycotoxin in the basolateral compartment at a certain time *t* [min]. The initial concentration in the apical compartment is *c*_0 h,ap_ and *V*_ap_ is the volume of the compartment at the beginning of the experiment. *A* is the surface of the filter insert membrane. As the polycarbonate membrane of the filter might slightly hinder the diffusion across the PBCEC monolayer and decrease the permeability, a cell-free transfer experiment was performed. A 100 μM mycotoxin solution was added to the apical compartment of a rat tail collagen coated Transwell^®^ filter insert. Samples were withdrawn after 30 min and quantified as described above. The resulting permeability of the mycotoxin across the cell monolayer *p*_c_ was calculated as shown in Eq 2:

$$\frac{1}{p_{\text{c}}[\text{cm}/\text{s}]}=\frac{1}{p_{\text{c}+\text{f}}[\text{cm}/\text{s}]}-\frac{1}{p_{\text{f}}[\text{cm}/\text{s}]}$$ (2)

Where *p*_c+f_ is the permeability coefficient of the transfer study and *p*_f_ the permeability coefficient of the cell-free polycarbonate filter membrane [9].

### Statistics

All data were statistically evaluated by Excel 2013 (Microsoft Corporation, Redmond, USA). Unpaired heteroscedastic Student’s T-test was used to calculate significant differences between data sets. Highly significant differences (p<0.001) are marked with ***, differences with medium significance (p<0.01) with ** and with low significance (p<0.05) with *.

The viability test was conducted in three different preparations each consisting of six individual replicates (*n* = 18). Transport studies including TEER and *c*_CL_ analysis were performed in three replicates in three independent preparations (*n* = 9). Studies aiming for the active transfer used three preparations with two replicates each (*n* = 6). ^14^C sucrose permeability was analyzed six times per filter in three replicates in one preparation (*n* = 18).

## Results

### Viability test

**Fig 2:**
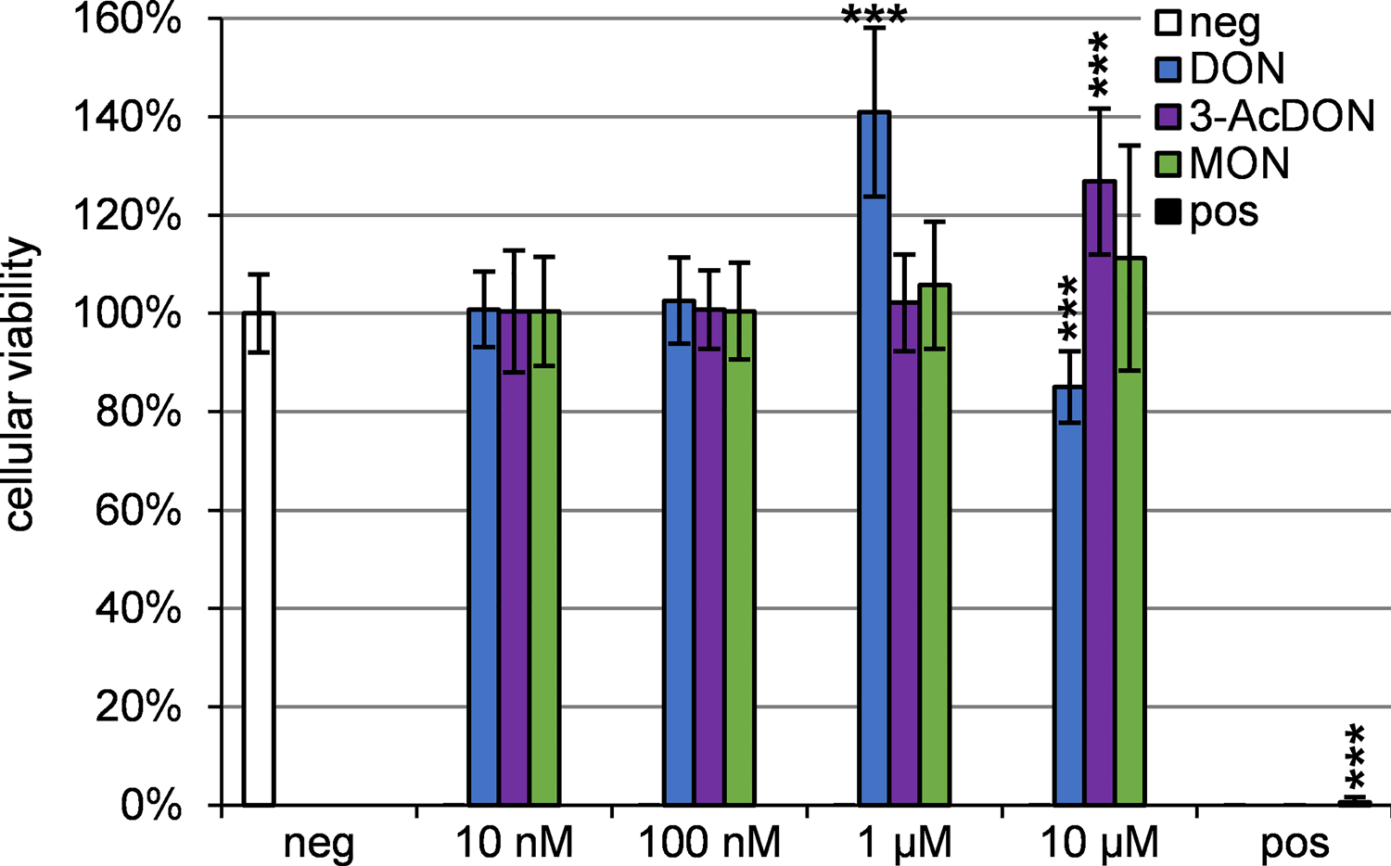
Cellular viability of PBCEC after 48 h incubation with DON, 3-AcDON and MON compared to the negative control analyzed with the CCK-8-assay (*n* = 18). Significant differences to the negative control are labeled. As positive control 10 μM T-2 toxin was used.

To determine the cytotoxic potential of the tested mycotoxins, the CCK-8-assay was performed using porcine brain capillary endothelial cells (PBCEC) which were incubated with 10 nM to 10 μM concentrations of the toxins for 48 h. The obtained results are shown in Fig 2.

The positive control 10 μM T-2 toxin showed a drastic reduction of the cellular viability to <2%, as described before [17]. For 10 μM DON a significant (*p*<0.001) reduction of the cellular viability to 85±7% was observed. The spindle-shaped PBCEC forming a confluent monolayer, partly detached from the collagen-coated bottom of the wells after 10 μM T-2 or DON incubation for 48 h h. Furthermore, PBCEC diameter was reduced, cell shape turned spherical and the monolayer revealed gaps in case of 10 μM DON incubation. After 10 μM T-2 incubation almost no cell-cell contacts were observed. DON concentrations of 1 μM as well as 10 μM 3-AcDON showed a significant increase in dehydrogenase activity, but no morphological changes compared to the negative control. MON showed no significant effects on the cellular viability and PBCEC.

### Barrier integrity

**Fig 3:**
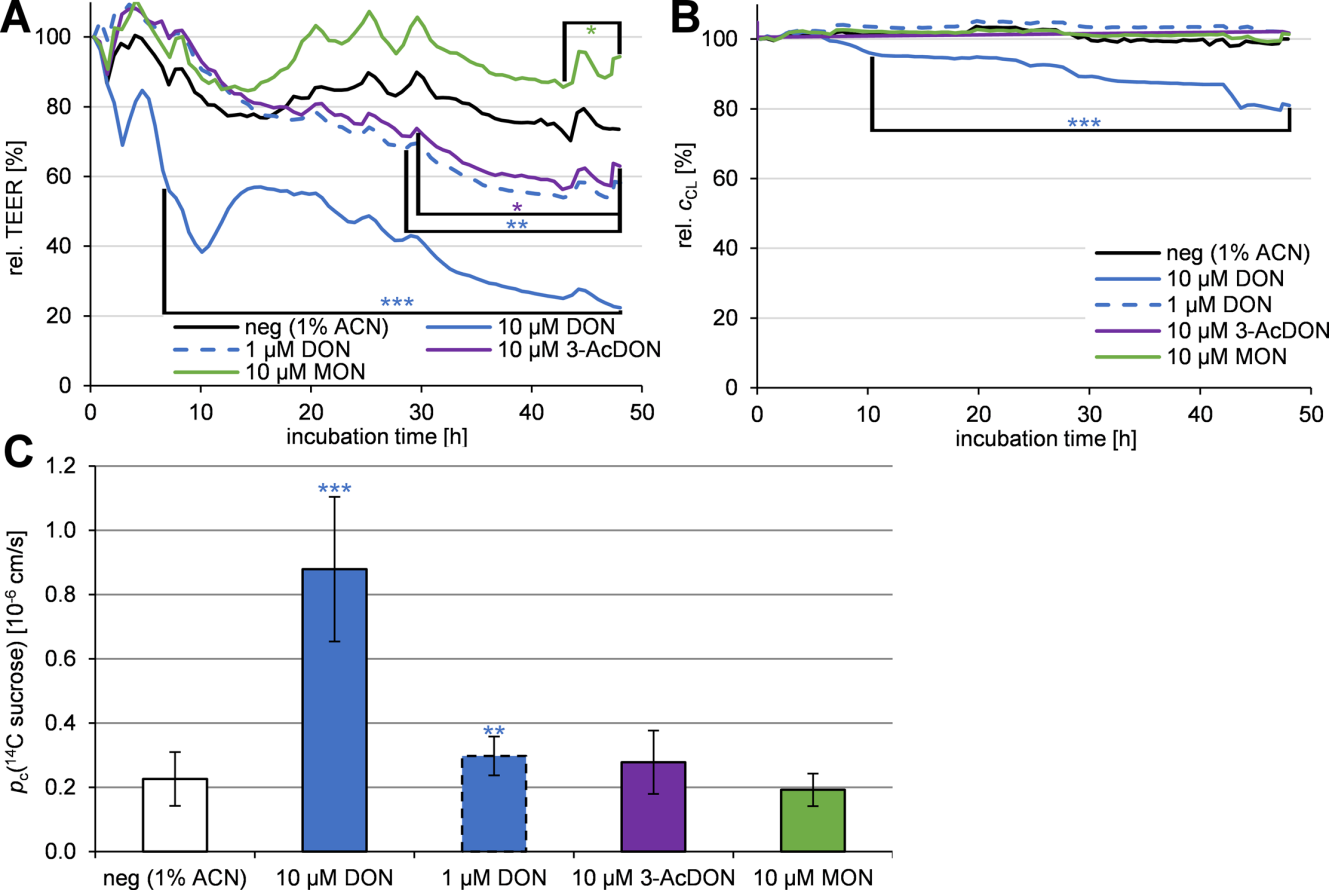
Relative TEER (A, *n* = 9) and electrical capacitance *c*_CL_ (B, *n* = 9) monitored for 48 h incubation after 10 μM toxin (DON: also 1 μM) incubation on PBCEC and sucrose permeability *p*_c_(^14^C sucrose) after 48 h (C, *n* = 18) using cellular impedance spectroscopy. Data are normalized to the TEER and *c*_CL_ at the beginning of the experiment. Average standard deviations for TEER: 6% (max. 15%); Average standard deviations for *c*_CL_ 2% (max. 7%). Error bars are not shown for the purpose of diagram clarity in A and B. Variations in A and B after 1, 2.5, 6.5, 18, 24, 28, 42, 48 h are due to sampling intervals for transfer studies. Significant differences in A and B between negative control and mycotoxin incubation are given, if differences maintained at least the given level of significance continuously until the end of the experiment. In diagram C DON, 3-AcDON and MON are compared to its solvent control containing 1% ACN, to determine significant differences.

Since MON and 3-AcDON showed no adverse effects on cellular viability, 10 μM concentrations were chosen for the incubation on Transwell^®^ filters. As DON showed a significant reduction of the cellular viability at 10 μM it was also incubated in concentrations of 1 μM to avoid a disruption of the cellular monolayer. TEER and electrical capacitance (*c*_CL_) values were recorded for incubation times of up to 48 h using cellular impedance spectroscopy and are shown in Fig 3A and 3B. To confirm the resulting TEER data, the permeability of ^14^C sucrose was analyzed after 48 h. Sucrose is no substrate of cellular transport proteins and serves as negative permeability marker in case of an intact BBB. The calculated permeability coefficients of ^14^C sucrose *p*_c_(^14^C sucrose) are given in Fig 3C.

As shown in Fig 3A 10 μM DON (blue line) reduces the TEER significantly (*p*<0.001) from 6.6 h to 48 h compared to the negative control (1% ACN, black line). After 48 h the TEER was reduced to 22±4%, whereas the negative control maintained at a TEER of 74±8%. The impairment of the barrier integrity was proven by the significant (*p*<0.001) increase in the permeability of ^14^C sucrose. Compared to the negative control with *p*_c_(^14^C sucrose) = 0.23±0.08×10^-6^ cm/s the permeability was increased by almost fourfold to *p*_c_(^14^C sucrose) = 0.88±0.23×10^-6^ cm/s after 48 h incubation with 10 μM DON (Fig 3C). When reducing the DON concentration to 1 μM, barrier integrity improved compared to 10 μM DON. The TEER was reduced continuously significant (*p*<0.01) compared to the negative control from 28.4 h until the end of the experiment (Fig 3A, blue dashed line). After 48 h the TEER dropped to 58±8%, which is clearly lower than the negative control, but also clearly higher than after 10 μM DON incubation. This effect could also be confirmed by a significant (*p*<0.01) increase in the permeability of ^14^C sucrose compared to the negative control resulting in *p*_c_(^14^C sucrose) = 0.30±0.06×10^−6^ cm/s. On the other hand, the barrier is clearly less permeable after 1 μM DON incubation compared to 10 μM DON incubation.

Similar results were obtained after incubation of 10 μM 3-AcDON. The TEER showed significant (*p*<0.05) differences to the negative control from 28.3 h until the end of the experiment. After 48 h the TEER was reduced to 63±9% (Fig 3A, purple line). The permeability reached *p*_c_(^14^C sucrose) = 0.28±0.10×10^−6^ cm/s, which is not significantly higher than the negative control. Therefore, 10 μM 3-AcDON caused a reduction in TEER, however, any impairment of the barrier was, in contrast to 10 μM DON, not strong enough to increase the permeability of sucrose relative to the negative controls.

When 10 μM MON were incubated, a significant (*p*<0.05) increase of the TEER was recorded between 43.7 h and 48 h. At the end of the experiment the TEER reached 94±15% (Fig 3A, green line). The permeability was *p*_c_(^14^C sucrose) = 0.19±0.05×10^−6^ cm/s, which is no significant change compared to the negative control. TEER and ^14^C sucrose permeability indicate almost no effects of MON on barrier integrity.

In addition to the TEER, cellular impedance spectroscopy was also used to monitor the electrical capacitance *c*_CL_ of the cell layer for 48 h as shown in Fig 3B. Since the cellular bilipid membranes act like an electrical capacitor, they also contribute to the cellular impedance. In contrast to the TEER, changes in *c*_CL_ are not related to the tight junctions, but to the integrity of the cell monolayer [45]. Weak cytotoxic effects result in a reduction of *c*_CL_, whereas due to the detachment of the cells strong cytotoxic effects are usually accompanied with an exponential increase of *c*_CL_ to more than 150%.

Continuously significant (*p*<0.001) changes of *c*_CL_ compared to the negative control were recorded after 9.5 h of incubation of 10 μM DON until the experiment was completed. After 48 h incubation of 10 μM DON the *c*_CL_ dropped to 81±2%, whereas the negative control maintained at 100±2%. Neither 3-AcDON, nor MON showed significant changes in the *c*_CL_.

### Transfer studies

**Fig 4:**
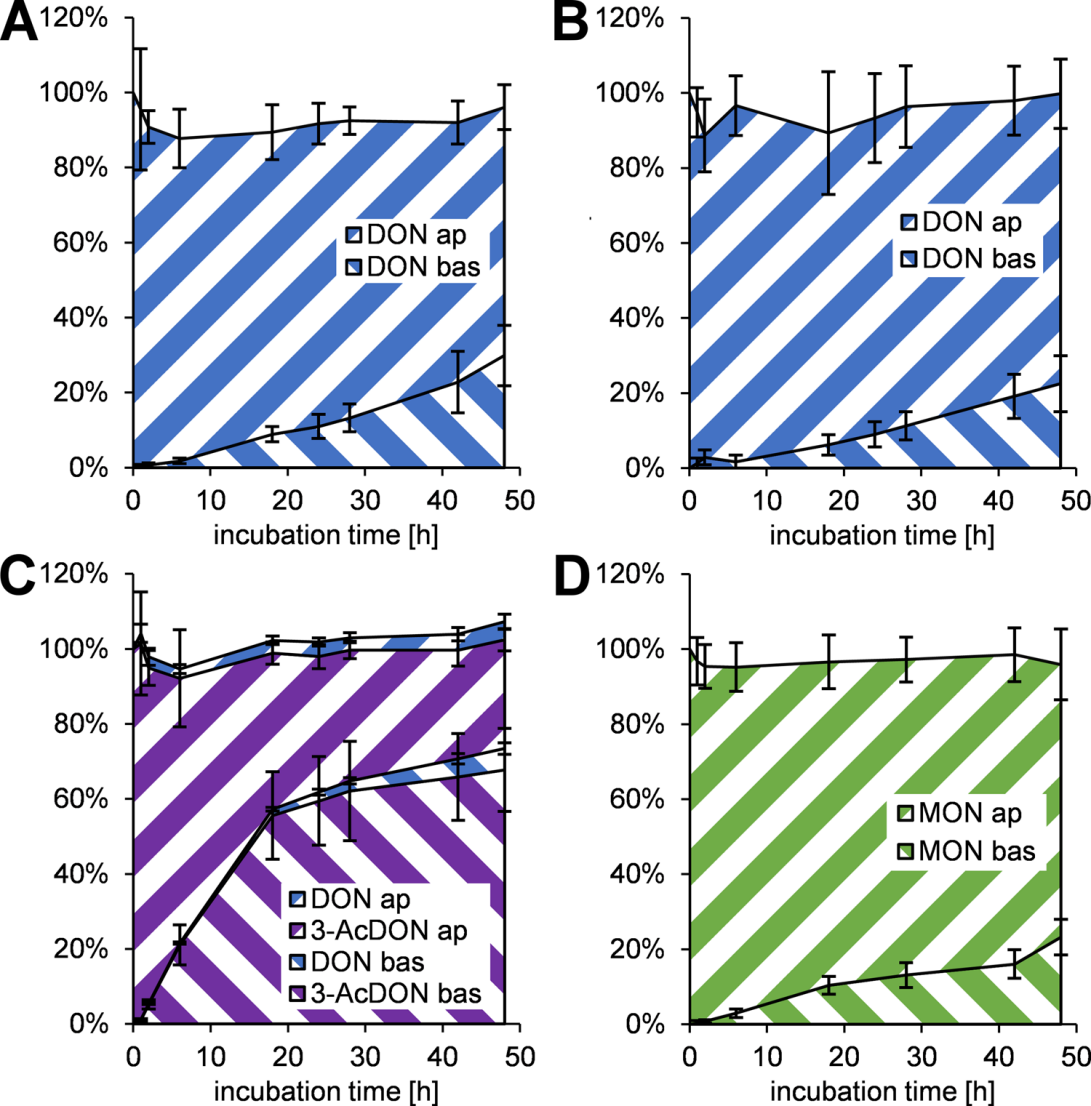
Transfer of 10 μM DON (A), 1 μM DON (B), 10 μM 3-AcDON (C), and 10 μM MON (D) to the basolateral (bas) compartment after application to the apical (ap) compartment of PBCEC in a time-dependent manner (*n* = 9). The distribution is normalized to the initial amount of substance (7.6 nmol, (B): 760 nmol) in the apical compartment. Areas shaded from bottom left to top right represent amounts of the toxin in the bas compartment, whereas areas shaded from top left to bottom right illustrate the amounts in the ap compartment. As the volumes of the apical and basolateral compartment vary, an equilibrium concentration between ap and bas is reached, at an ap/bas amount ratio of 32%/68%, which corresponds to a volume ratio of 0.76 mL/1.65 mL.

As shown in the previous section, the used mycotoxin concentrations (1–10 μM) might lead to an impairment of the BBB, but not to a breakdown of the barrier. This is an important prerequisite for performing transfer studies. In case of a BBB breakdown, compounds would permeate much faster through the cell layer. To investigate the fate and distribution of 10 μM (DON: also 1 μM) apically applied mycotoxins, DON and 3-AcDON were analyzed at eight points in time up to 48 h by HPLC-MS/MS, whereas MON was analyzed by HPLC-HRMS. The results are summarized in Fig 4. To receive comparable data, permeability coefficients were calculated according to (1) and (2) and summarized in Table 1.

**Table 1:** Permeability coefficients of the tested mycotoxins compared to the negative permeability marker ^14^C sucrose from the apical to the basolateral compartment of a PBCEC monolayer (*n* = 9).

compound	*p*_c_ [10^-6^ cm/s]	*t* [h]
**^14^C sucrose**	0.23±0.08	0.17–1.33
**10 μM DON**	1.18±0.31	48
**1 μM DON**	0.88±0.29	48
**10 μM 3-AcDON**	6.67±1.53	6.5
10 μM 3-AcDON incl. DON*	6.85±1.60	6.5
**10 μM MON**	1.07±0.23	48

Points in time *t* were chosen at transfer rates <20% (referring to the concentrations), to exclude rediffusion effects and therefore vary between the compounds.

(* indicates that *p*_c_ of 3-AcDON was calculated including the formed metabolite DON).

Fig 4A (10 μM DON) and Fig 4B (1 μM DON) show, how the continuous increase of DON in the basolateral compartment was accompanied with a continuous decrease of apical concentrations in the same relation. Furthermore, the total recovery was around 100% at all incubation times, indicating that DON metabolites play a negligible role in the distribution of DON at the BBB. After 48 h 66±6% of the apical applied DON (10 μM) were recovered in the apical compartment and 30±8% were detected in the basolateral compartment (Fig 4A). From this data a permeability coefficient of *p*_c_(DON) = 1.18±0.31×10^-6^ cm/s was calculated. After 48 h application of 1 μM DON the transfer to the basolateral compartment was slightly slower. As shown in Fig 4B 77±9% of the administered DON were recovered in the apical and 22±8% in the basolateral compartment, resulting in a permeability coefficient of *p*_c_(DON) = 0.88±0.29×10^-6^ cm/s.

The transfer rates of 10 μM 3-AcDON were much faster compared to DON and reached an almost equal distribution between both compartments after 18 h (Fig 4C). In contrast to all other mycotoxins tested in this study, 3-AcDON underwent metabolism by cellular enzymes. Due to the cleavage of the 3-acetylester, the resulting metabolite DON has to be taken into account, when analyzing the transfer rates of 3-AcDON. After 18 h only 42±3% 3-AcDON and 3±1% DON were detectable in the apical compartment, whereas 56±12% 3-AcDON and 2±0% DON could be recovered in the basolateral compartment. Besides the time-dependent accumulation of DON due to longer incubation time after 48 h this distribution underwent only minor changes compared to the distribution at 18 h. After 48 h 29±3% 3-AcDON and 5±2% DON were detected in the apical compartment, whereas 68±11% 3-AcDON and 6±2% DON were recovered in the basolateral compartment (Fig 4C). At each point in time almost 100% of the applied mycotoxin were recovered, therefore it is concluded that other 3-AcDON metabolites than DON are negligible. As long as referring to 3-AcDON separately, a permeability coefficient of *p*_c_(3-AcDON) = 6.67±1.53×10^-6^ cm/s was calculated. If the metabolite DON is included, the permeability coefficient raises to *p*_c_(3-AcDON+DON) = 6.85±1.60×10^-6^ cm/s.

Fig 4D shows the distribution of 10 μM MON initially applied in the apical compartment. The transfer rate to the basolateral compartment was similar to the transfer of DON presented in Fig 4A and 4B. After 48 h 76±12% MON were recovered in the apical compartment, whereas 27±6% MON were detectable in the basolateral compartment. From these data a permeability coefficient of *p*_c_(MON) = 1.07±0.23×10^-6^ cm/s was calculated.

In addition to the quantification in apical and basolateral cell culture media, the filter membranes including the PBCEC were extracted and analyzed after 48 h of incubation (data not shown). Although most mycotoxins could be detected in these filter extracts, their amounts in relation to the initially applied amounts were below 1%. Therefore, the intracellular amounts of the mycotoxins are negligible in terms of contributing to the total distribution of mycotoxin in the system.

### Active transfer studies

**Fig 5:**
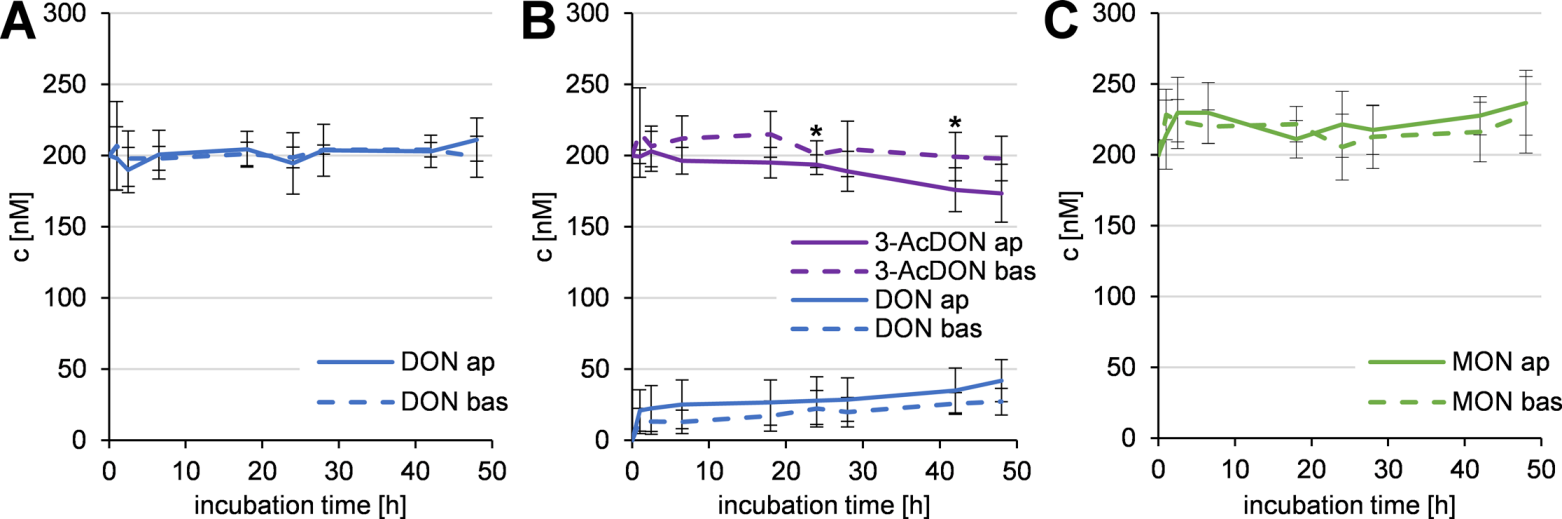
Recovery of 200 nM DON (A), 200 nM 3-AcDON (B), and 200 nM MON (C) applied to the apical and basolateral compartment in a time-dependent manner (*n* = 6) on a PBCEC monolayer. Due to the different volumes of both compartments, the sum of both compartments does not yield 400 nM in total at all given points in time. Significant differences between mycotoxin concentrations in the apical (ap) and basolateral (bas) compartment are labeled.

In contrast to the transfer studies discussed above, the mycotoxins were also applied in both compartments in equimolar concentrations of 200 nM, to investigate an enrichment in the apical or basolateral compartment, which would indicate an active transport. The mycotoxin concentrations were analyzed as described above at eight points in time up to 48 h by HPLC-MS/MS or HPLC-HRMS. The results are shown in in Fig 5.

For DON, shown in Fig 5A, no enrichment in the apical or the basolateral compartment was detected. After 48 h 211±15 nM DON was detected in the apical and 199±14 nM DON in the basolateral compartment.

Results of 3-AcDON incubation are shown in Fig 5B. As 3-AcDON is hydrolyzed in the used cells, the resulting metabolite DON was also analyzed. 48 h after the equimolar application of 200 nM 3-AcDON in both compartments 174±20 nM 3-AcDON and 42±15 nM DON were detectable in the apical compartment. In the basolateral compartment 198±16 nM 3-AcDON and 27±9 nM DON were recovered. The differences between apical and basolateral compartment for 3-AcDON reached statistical significance (*p*<0.05) only after 18 h and 42 h whereas the differences for DON were not statistically significant at any point in time. As no significant differences were retained continuously until the end of the experiment, an active transfer or a noteworthy enrichment of DON or 3-AcDON can be excluded.

After 48 h equimolar incubation of 200 nM MON in both compartments 237±23 nM MON were detected in the apical and 228±27 nM MON in the basolateral compartment (Fig 5C). No significant differences between apical and basolateral values were observed at any point in time up to 48 h indicating that MON is no substrate of an efflux transport protein and therefore not enriched in any compartment.

## Discussion

### DON

DON reduced cellular viability (Fig 2) and impaired the barrier integrity drastically at concentrations of 10 μM (Fig 3A). Furthermore, 10 μM DON increased the permeability of ^14^C sucrose (Fig 3C) and caused alterations in the cellular membrane integrity (Fig 3B). This indicates distinct effects on tight junctions even at 1 μM, but only minor cytotoxic effects. However, a complete loss of the barrier function was not observed. In Caco-2 cells DON increases the cellular inflammation markers such as MAPK. MAPK are known to reduce the expression of claudins, which are important to maintain the performance of tight junctions [19]. A similar mechanism might exist for DON at the BBB, leading to reduced barrier integrity and therefore increased permeability. Compared to previous results with T-2 and HT-2 toxin, both type A trichothecenes showed similar effects, but due to their higher cytotoxicity already at nanomolar concentrations [17]. Since DON is one of the most frequently detected mycotoxins in European food and feed, the human exposure has to be considered as high. Previous studies analyzing urinary human mycotoxin biomarkers found the TDI of DON of 1 μg/kg bodyweight exceeded in many cases indicating the relevance of DON concerning human health [25, 26, 24, 46]. With regard to the effects on the BBB, it is important to take the high occurrence in food and considerably high oral bioavailability of DON into account [28, 27, 47]. The cytotoxicity of DON combined with weakening effects of barrier and membrane integrity make DON the potentially most critical mycotoxin tested in this study. The observed effects are likely to occur especially after very high DON intake or after long-term exposure to DON. However, the applied concentrations in this static *in vitro* model cannot be directly compared to the dynamic situation *in vivo*. The DON uptake to the brain compartment was of moderate speed (Fig 4A). Application of 1 μM DON caused less adverse effects to the PBCEC monolayer (Fig 3) and showed similar transfer rates (Fig 4B). Due to its low lipophilicity, it remained unclear so far, whether DON could cross the BBB. However, the calculated permeability coefficients show a four to five times higher transfer rate of DON to the brain compared to the negative control ^14^C sucrose (Table 1). The permeability coefficient is in the same order of magnitude as morphine in this cell culture model [48]. Morphine is known to cross the BBB, but due to its low lipophilicity, morphine does not reach the brain as fast as other CNS-active xenobiotics. In this study no enrichment in one of the compartments was detected (Fig 5A), which indicates that DON is not a substrate of a major transport protein. Earlier studies suggested the same, because inhibitors for Pgp and MRP did not alter the transfer rates of DON in Caco-2 cells [21].

### 3-AcDON

3-AcDON, an often co-occurring acetylated DON derivative, was tested in this study for its effects on the BBB and to compare it with DON and the already analyzed type A trichothecenes T-2 and HT-2 toxin [17]. Using the CCK-8 assay an increase in dehydrogenase activity was detected (Fig 2). Since 3-AcDON did not reduce the cellular viability, these effects might not be cytotoxic, because no morphological changes were detected as they were observed after 10 μM DON incubation. Alterations in barrier integrity were detected (Fig 3A), but only with low statistical significance and could not be confirmed by significant changes in ^14^C sucrose permeability (Fig 3C). The cellular membrane integrity was unaffected by 10 μM 3-AcDON incubation for 48 h (Fig 3B). Compared to 15-AcDON and DON, 3-AcDON is less capable of increasing cellular inflammation markers such as MAPK in Caco-2 cells [19]. MAPK have been shown to reduce claudins, the major tight junction proteins maintaining the barrier integrity in the intestinal barrier as well as the BBB. These explanations are consistent with the results in these studies, since reduced MAPK levels after incubation of 3-AcDON compared to DON should result in less cytotoxic and especially less barrier weakening effects [20]. On the one hand the cytotoxic and barrier impairing effects of DON and 3-AcDON on PBCEC were clearly weaker compared to the already published effects of the type A trichothecenes T-2 toxin and HT-2 toxin. T-2-toxin caused stronger effects at nanomolar concentrations than 10 μM DON [17]. On the other hand DON is a more common contaminant in food and feed and human exposure to DON is higher compared to T-2 toxin [49]. The transfer rates and permeation coefficients of 3-AcDON were approximately six times higher than for DON (Fig 4A and 4C, Table 1) and clearly the fastest for any of the tested mycotoxins in this study. Therefore, 3-AcDON can very likely permeate the BBB and reach neuronal tissues. The fast permeation of 3-AcDON across the BBB can be explained by its acetylation at C-3 (Fig 1) strongly increasing the lipophilicity compared to DON. Besides the fast 3-AcDON transfer, a hydrolysis of the ester at C-3 was observed (Fig 4C), releasing the unconjugated and more toxic DON. Neither 3-AcDON nor DON as hydrolysis product were continuously enriched in one of the compartments after equimolar incubation of 3-AcDON in both compartments (Fig 5B). This leads to the conclusion that neither 3-AcDON nor DON are substrates of efflux transport proteins. Despite it has not yet been detected in mammalian circulation after oral administration, it cannot be ruled out that 3-AcDON is of toxicological importance since it is capable of permeating the BBB easily and releasing the more toxic DON at both sides of the BBB.

### MON

MON belongs to the diverse group of so called emerging mycotoxins. Although MON has been known for some decades, only a limited number of studies dealing with the toxicity have been performed [50, 51]. A possible reason for this might be that MON is difficult to analyze and quantify with conventional methods. To overcome problems concerning MON analysis, an optimized HPLC-HRMS method was used allowing the quantification of MON levels down to 50 nM in cell culture samples [36]. The incubation of 10 μM MON did not affect the cellular viability of PBCEC up to 48 h (Fig 2). Furthermore, MON does neither affect the integrity of cellular membranes (Fig 3B) nor increase the permeability of the PBCEC monolayer to ^14^C sucrose (Fig 3C). A slight increase of the barrier integrity was detectable at the very end of the experiments (Fig 3A), but should be considered as negligible considering low statistical significance. On the one hand, MON is a very small molecule (Fig 1), enabling it to easily permeate through barrier forming tissues. On the other hand, it is also a very strong acid, which is highly polar and therefore hardly permeating cellular membranes. The detected transfer rate of MON across the BBB in this study (Fig 4D) is within the same order of magnitude as DON (Fig 4A and 4B, Table 1) and comparable to the CNS-active drug morphine [48]. MON was not enriched in one of the compartments after equimolar application in both compartments (Fig 5C), leading to the assumption that MON is not an efflux transporter substrate. Since MON permeates the BBB to some extent and exhibits a considerably high bioavailability *in vivo* [38, 39], it might cause effects on neuronal tissues. It is reported that MON inhibits pyruvate and α-ketoglutarate dehydrogenases in 5 μM concentrations [37]. Since the brain contributes to 2% of the total human body mass, but consumes 20% of the total energy, it can be considered a vulnerable target for MON and its effects on energy metabolism. Still it should be clarified, whether these effects also occur *in vivo* and contribute to MON toxicity. Nevertheless, the strong discrepancy of MON *in vivo* and *in vitro* toxicity still remains unclear and is an interesting field for further research [38, 39, 51].

## Conclusion

In this study data on the effects of mycotoxins on the BBB is presented. PBCEC are a well-established model to study these effects *in vitro* [8, 9]. Although the results may not be transferred directly to the effects on the complex and dynamic human brain *in vivo*, they provide valuable information for research focusing on the potential neurotoxicity of mycotoxins.

Taken together, it can be summarized that trichothecenes (especially DON) could cause cytotoxic effects at the BBB and reduce its integrity. 3-AcDON and MON were shown to exhibit much weaker or almost no adverse effects at the BBB *in vitro*. Referring to their transport properties, these are generally driven by polarity and molecular size of the mycotoxin. DON is a hydrophilic molecule of medium molecular size, whereas MON is very small, but also very polar. DON and MON were transferred three to four times faster across the PBCEC monolayer than the negative control ^14^C sucrose and reached permeabilities comparable to morphine, which is a CNS-active and BBB permeable drug [48]. This is in agreement with their polarity and molecular size. In conclusion the results of this study suggest that DON and MON are BBB permeable mycotoxins, although to a limited extent. Finally, 3-AcDON was transferred approximately four times faster than DON and MON. Although it is slightly larger than DON, the acetylation considerably increases the lipophilicity of 3-AcDON leading to a high BBB permeability. 3-AcDON is very likely to cross the BBB, and release the more toxic DON by ester hydrolysis. For this reason, it should not be neglected in toxicity assessment and risk evaluation.

Considering the presented strong differences in their effects at the BBB, future research should investigate the effects of further mycotoxins on the BBB, to obtain a better overview of their effects on the cerebral tissues. In addition, the common co-occurrence of *Fusarium* mycotoxins in food and feed might lead to combination effects. In case of the BBB, trichothecenes could impair the integrity of the BBB and allow other mycotoxins, which would not pass the BBB as single compound, to reach the brain.
